# An Internal Review of Rates of Palliative Medicine Referral for Patients With Advanced Pancreatic Cancer

**DOI:** 10.7759/cureus.19670

**Published:** 2021-11-17

**Authors:** Deanna L Huffman, Urwat T Vusqa, Karthik Shankar, Lynna Alnimer, Yazan Samhouri, Srividya Srinivasamaharaj, Srikrishna V Malayala, Dulabh Monga

**Affiliations:** 1 Internal Medicine, Allegheny Health Network, Pittsburgh, USA; 2 Internal Medicine, Ascension Providence Hospital, Southfield, USA; 3 Department of Hematology and Cellular Therapy, Allegheny Health Network Cancer Institute, Pittsburgh, USA; 4 Hematology, Medical Oncology, Piedmont Healthcare, Columbus, USA; 5 Internal Medicine, Temple University Hospital, Philadelphia, USA; 6 Department of Hematology and Oncology, Allegheny Health Network Cancer Institute, Pittsburgh, USA

**Keywords:** electronic health record, quality review, pain management, palliative care, pancreatic cancer

## Abstract

Background

The American Society of Clinical Oncology recommends that patients with advanced cancer receive palliative care services in concurrence with active treatment. While the benefits of palliative care are clear, integration of palliative care can be challenging. We aim to review rates of palliative care consultation in patients with advanced pancreatic cancer at our institution, intending to improve these rates.

Methods

We retrospectively reviewed the electronic records of all patients with pancreatic cancer treated at Allegheny General Hospital diagnosed between 2009-2020. Summary statistics are presented as percentages for categorical data and median with interquartile range for quantitative data.

Results

Of the 171 patients reviewed, 121 completed all treatment and evaluation within our health network (Pittsburgh, United States). The median age was 63 years (IQR 40-91 years); 55 patients (45%) were male; the majority were white (107 patients, 88%). At the time of diagnosis, 28% of our patients had stage IV disease (34 patients), and 19.8% of patients who developed stage IV disease had palliative care referrals.

Conclusions

Palliative care is an integral part of usual care for advanced pancreatic cancer. Our analysis showed that palliative care is underutilized in our hospital. We aim to improve palliative care integration in our patients’ care by adding a hard stop to electronic medical records to remind physicians to offer palliative care to our patients with pancreatic cancer and to arrange lecture series to emphasize the importance of palliative care in this setting.

## Introduction

Pancreatic cancer is the fourth leading cause of cancer-related death in the developed world and seventh worldwide [1]. It is associated with high mortality, with median survival of 4.6 months and overall survival of 3% at five years [2-3]. The poor outcomes are attributed to non-specific symptoms, leading to a delay in diagnosis [1-3]. Moreover, it is associated with aggressive tumor biology and early spread [1-3]. The treatment of pancreatic cancer includes radical resection of the pancreas with adjuvant chemotherapy [1]. However, only 20% of those initially diagnosed are eligible for the definitive treatment, as the remaining 80% present with advanced disease [1-3]. Even with complete resection, the prognosis remains grim; for node-negative disease, the five-year survival after pancreaticoduodenectomy is ∼ 25-30% and for node-positive disease, the five-year survival drops to 10% [1-4].

As pancreatic cancer is usually diagnosed at an advanced stage, patients often suffer from a high symptom burden and associated emotional stressors. Bothersome symptoms can be wide-ranging and include pain, jaundice with associated pruritus, ascites, pancreatic exocrine insufficiency leading to digestive difficulty, pancreatic endocrine insufficiency leading to diabetes, gastric outlet obstruction, anorexia, and weight loss [5-6]. The diagnosis of pancreatic cancer is also associated with the development of anxiety, depression, and sleep disturbances, and patients diagnosed with pancreatic cancer are more likely to commit suicide than the general population [7-9].

The integration of palliative care services into the treatment of pancreatic cancer can help patients reduce pain, improve quality of life, and has even been shown to prolong survival [4-5]. Several randomized controlled trials have been completed in the past decade, which confirm these benefits, and the American Society of Clinical Oncology (ASCO), the American Cancer Society, and the World Health Organization all recommend providing palliative care interventions simultaneously with treatment for advanced cancer [10-13].

Despite the proven benefit of early palliative care involvement, integration of palliative care into standard oncologic treatment for pancreatic cancer is not always straightforward. Referral to a specialized palliative care service can be delayed because of the patients’ and physicians’ misconception about palliative care being an alternative or non-traditional care strategy [13]. Additionally, there may be geographical barriers, such as inadequate hospice care, in more rural areas [14]. Availability of palliative care services has also become an issue as the field expands faster than providers can be trained and the availability of palliative care services and training varies widely [15-16].

Oncologists and palliative care specialists have a shared responsibility to openly discuss prognosis and recommend end-of-life care options at appropriate times in a cancer patient’s disease course. The aim of this study is to assess how frequently were patients with pancreatic cancer referred to palliative care service at our institution with the aim of improving patient outcomes.

The abstract of this research was submitted for the annual ASCO Meeting 2021, and the abstract was published online in the Journal of Clinical Oncology, volume 39, 2021.

## Materials and methods

We retrospectively reviewed the electronic medical records of all patients diagnosed with pancreatic cancer at Allegheny Health Network from 2009-2020. Patients with significant missing demographic or follow-up data were excluded. Demographic information was collected from admissions face sheets. Clinical staging data is in compliance with the American Joint Committee on Cancer 8th edition staging system [17]. Information on pain was collected from the initial visit history and physical exam, and pain was rated by the patient on a scale of 1-10 with 1 being “minimal pain” and 10 being “the worst pain I’ve ever felt.” Records were reviewed for whether the patient was referred to palliative care services at any time, in the inpatient or outpatient settings, prior to their death or last contact. Summary statistics are presented as absolute numbers and percentages for categorical variables and median with interquartile range for continuous variables. To assess attitudes on palliative care referral within the cancer center, surveys were sent to all hematology and oncology fellows and staff. There was a 40% response rate for fellows and a 30% response rate for attending physicians. The study was exempted by the institutional review board of Allegheny Health Network.

## Results

Of the 171 patients reviewed, 121 completed the entire treatment and evaluation within our health network. The baseline characteristics are outlined in Table 1. The median age was 63 years (IQR 40-91 years); 55 patients (45.5%) were male; the majority were white (107 patients, 88.4%). At the time of diagnosis, 28.1% of our patients (34 patients) had stage IV disease. Following patients through 2020, 86 patients (71%) eventually developed stage IV disease, and 19.8% of those patients with documented stage IV disease had documented palliative care referrals in the outpatient settings provided by oncologists. Eight patients had pain documented at diagnosis, six patients underwent celiac plexus neurolysis, 31 underwent palliative radiation and 43 patients had documented opiate use.

**Table 1:** Baseline characteristics for all patients diagnosed with pancreatic cancer at Allegheny General Hospital from 2009-2020

Characteristic	Number of patients (%) (n = 121)
Median Age, y	63.0
Gender	
Male	55 (45.5%)
Female	66 (54.5%)
Race	
White	107 (88.4%)
Black	11 (9.0%)
Non-Black Hispanic	1 (0.8%)
Other	2 (1.7%)
Stage at Diagnosis	
I	48 (39.7%)
II	29 (24.0%)
III	10 (8.3%)
IV	34 (28.1%)
Patients Reporting Pain at Diagnosis	88 (72.7%)
Patients Listed as Stage IV by 2020	86 (71.0%)
Palliative Referral	24/86 (27.9%)
No Palliative Referral	62/86 (72.1%)

Palliative interventions and associated pain ratings are shown in Table 2. Of the six patients who underwent celiac plexus neurolysis, only four documented pain at the six-month follow-up. Of the 31 patients who underwent palliative radiation, six documented pain at the six-month follow-up.

**Table 2:** Number of patients who received either celiac plexus neurolysis or radiation with palliative intent as part of treatment for advanced pancreatic cancer

Palliative Intervention	Patients with Pain at Diagnosis (No.)	Patients with Pain at 6 Months (No.)
Celiac Plexus Neurolysis	6	4
Radiation	31	6

## Discussion

Although the benefits of the early integration of palliative care into the treatment of pancreatic cancer have been demonstrated, the creation of a system whereby providers can be adequately trained in palliative care and sufficient palliative care recourses are available has not yet been achieved. The results of our internal review reflect the need for our program to create a more unified approach to palliative care.

As the support behind the integration of palliative care services into the standard care of patients diagnosed with pancreatic cancer continues to grow, barriers are constantly being identified. We prefer to think of barriers to the successful integration of palliative care as either oncologist-driven, patient-driven, or system-driven. Oncologist-driven factors include inadequate knowledge about palliative care or negative attitudes toward palliative care services. A 2012 survey of 603 Canadian medical oncologists, radiation oncologists, and surgical oncologists revealed that 84% of respondents referred terminally ill patients to palliative care but only 37% were aware that patients actively receiving chemotherapy could be referred to palliative care services; interestingly, one-third of respondents reported they would have referred to palliative care earlier if it was renamed “supportive care [18].” A 2015 survey of 182 oncologists by Hui et al. again found the name “supportive care” to be preferred and that the name “palliative care” was more likely to be synonymous with “hospice” (PC, 53% vs. SC, 6%; p < .001) [19]. These findings may suggest that oncologists are uncomfortable with the term “palliative care” and could be less likely to refer because they find it incongruent with active treatment. Another oncologist-driven factor may be inadequate training in the field. A review of 254 second-year oncology fellows in the United States showed that only 26% had completed a palliative care rotation; when tested on basic palliative care knowledge, the fellows scored a median of 2 of 4, and only 23% could correctly perform an opiate conversion [20].

Patients themselves are often resistant to the idea of palliative care. A 2016 trial by Zimmerman et al. randomized patients with advanced cancer who received either standard care or early palliative care and then surveyed both patients and caregivers about attitudes toward palliative care. Initial perceptions in both trial arms about palliative care included associations with death, comfort care, or hopelessness [21]. A common theme was that palliative care was synonymous with death and with care in the last weeks of life [21].

Finally, systems-driven factors include inadequate availability of palliative care resources or lack of infrastructure to support timely access to these resources. A 2010 survey review by Hui et al. showed that while 98% of National Cancer Institute (NCI)-designated cancer centers had a palliative care program, only 78% of non-NCI centers had such a program (P = 0.002); NCI centers were also much more likely to have an outpatient palliative care clinic (59% vs 22%, P<0.001) [16]. As NCI-designated cancer centers represent about 4% of all cancer treatment centers in the United States, there may be many patients who simply do not have access to these resources due to geographical location [22].

**Figure 1:**
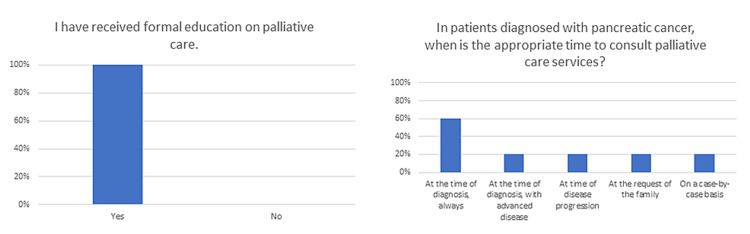
Selected survey responses are shown with the question-and-answer choices as they were presented in the survey. Data are presented as a percentage of respondents.

**Figure 2:**
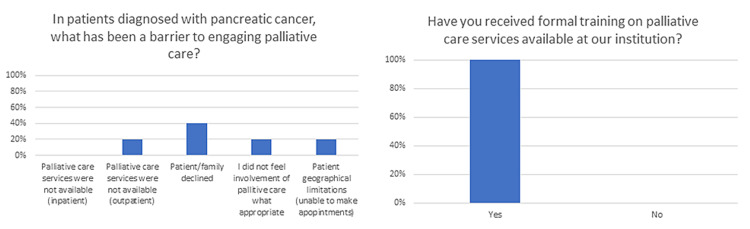
Continued: Selected survey responses are shown with the question-and-answer choices as they were presented in the survey. Data are presented as a percentage of respondents.

To further investigate roadblocks to the successful integration of palliative care within our own program, we performed an internal survey of fellows and facilities to gauge attitudes toward palliative care. Selected survey responses are shown in Figures 1-2. While this survey is not internally validated, we felt it helpful in guiding our evaluation of the integration of palliative care services at our institution. Selected survey responses are shown in Figure 1. While 100% of respondents said they had received formal training on palliative care services, only 60% recognized that palliative care services should be involved at the time of diagnosis of pancreatic cancer. Identified barriers included the unavailability of palliative care services (20%), palliative care services were offered but declined by the patient or family (40%), and palliative care services were available but felt to be inappropriate (20%).

The limitations of this study include its small sample size. We evaluated 171 patients within one community-based cancer center in the outpatient setting. Our results may be difficult to extrapolate to larger populations or to other hospital systems and cancer treatment centers. We also recognized our results may be open to bias due to missing data, as our records did not have consistently recorded pain ratings with each visit, which can make these results difficult to interpret.

Moving forward, we hope to improve palliative care integration in our patients’ care by adding a hard stop to electronic medical records to remind physicians to offer palliative care to our patients with pancreatic cancer at the time of diagnosis. We also plan to arrange medical grand rounds and various lectures to increase awareness of the importance of palliative care in this setting.

## Conclusions

The integration of palliative care is a vital part of usual pancreatic cancer treatment and current treatment guidelines reflect this. In our program, we fall short of providing timely palliative care consultation to our patients diagnosed with pancreatic cancer. An internal survey revealed a lack of oncologist education, patient education, and resource availability as barriers to successful palliative referral. We hope to use this information to further refine our practices and provide adequate palliative care integration to our patients with pancreatic cancer.
